# Biomechanical comparison of plantar-to-dorsal and dorsal-to-plantar screw fixation strength for subtalar arthrodesis

**DOI:** 10.31744/einstein_journal/2020AO5052

**Published:** 2020-02-27

**Authors:** Nileshkumar Chaudhari, Alexandre Leme Godoy-Santos, Cesar de Cesar Netto, Ramon Rodriguez, Shouchen Dun, Jun Kit He, Haley McKissack, Glenn S. Fleisig, Eduardo Araujo Pires, Ashish Shah

**Affiliations:** 1 University of Alabama at Birmingham BirminghamAL United States University of Alabama at Birmingham , Birmingham , AL , United States .; 2 Hospital Israelita Albert Einstein São PauloSP Brazil Hospital Israelita Albert Einstein , São Paulo , SP , Brazil .; 3 Hospital das Clínicas Faculdade de Medicina Universidade de São Paulo São PauloSP Brazil Hospital das Clínicas , Faculdade de Medicina , Universidade de São Paulo , São Paulo , SP , Brazil .; 4 University of Iowa Health Care Carver College of Medicine Iowa CityIowa United States University of Iowa Health Care , Carver College of Medicine , Iowa City , Iowa , United States .; 5 Tulane University Orthopaedics New OrleansLA United States Tulane University Orthopaedics , New Orleans , LA , United States .; 6 American Sports Medicine Institute BirminghamAL United States American Sports Medicine Institute , Birmingham , AL , United States .

**Keywords:** Subtalar joint, Arthrodesis, Ankle, Joint instability, Arthritis, Joint diseases

## Abstract

**Objective:**

To compare screw fixation strength for subtalar arthrodesis.

**Methods:**

Eight matched pairs of cadaver feet underwent subtalar joint arthrodesis with two 7.3mm cannulated screws. Randomization was used to assign screw orientation, such that one foot in each pair was assigned dorsal to plantar screw orientation (DP Group), and the other foot, plantar to dorsal orientation (PD Group). Standard surgical technique with fluoroscopy was used for each approach. Following fixation, each specimen was loaded to failure with a Bionix ^®^ 858 MTS device, applying a downward axial force at a distance to create torque. Torque to failure was compared between DP and PD Groups using Student’s *t* test, with p=0.05 used to determine statistical significance.

**Results:**

Statistical analysis demonstrated that the mean torque to failure slightly favored the DP Group (37.3Nm) to the PD Group (32.2Nm). However, the difference between the two groups was not statistically significant (p=0.55).

**Conclusion:**

In subtalar arthrodesis, there is no significant difference in construct strength between dorsal-to-plantar and plantar-to-dorsal screw orientation. The approach chosen by the surgeon should be based on factors other than the biomechanical strength of the screw orientation.

## INTRODUCTION

Arthrodesis of the subtalar joint is an effective treatment for patients with isolated subtalar arthritis or instability. ^( 1 , 2 )^ Subtalar joint arthritis may develop idiopathically or secondary to trauma ^( 3 )^ inflammatory arthropathy, ^( 4 )^ tarsal coalition, ^( 5 )^ and ankle arthrodesis. The subtalar joint may become unstable by ligament or tendon insufficiency, ^( 6 , 7 )^ or by neuromuscular dysfunction. ^( 8 )^ Subtalar arthrodesis is done as a component of triple arthrodesis for stage three adult-acquired flat foot to correct hind foot misalignment. However, for talocalcaneal conditions, isolated subtalar arthrodesis has also been advocated for with cited advantages of lower risk of adjacent joint arthritis and lower risk of nonunion or malunion of the transverse tarsal joint. In all cases the procedure is performed to relieve pain and improve function. ^( 1 , 2 , 9 )^ The root of this pain lies in changes in articular geometry and, therefore, joint motion, in these pathologies. There is increased stress on joints in the area and the foot is forced to perform abnormal motions, resulting in increased contact stress and forces that cause further degeneration. ^( 10 )^ The goal is to fuse the subtalar joint in a solid and physiologic position to stop further painful motion. ^( 11 )^

Fusion rates in subtalar arthrodesis range from 84% to 100% in various studies. ^( 1 , 2 , 12 )^ Operative techniques have included various adjuncts to help achieve fusion, including autogenous bone graft, allograft, and, more recently, various orthobiologics. ^( 13 )^ Subtalar arthrodesis with autologous bone grafts and secured with cancellous bone screw showed ample stability and rate of union. Prior to 1970, internal fixation was not used for these procedures, particularly in children. Initially, Steinmann pins were used to maintain the achieved position of the joint, and later staples and power devices were used. ^( 14 , 15 )^ With the advent of AO techniques (https://www.aofoundation.org/), compression screws were introduced to maintain position and to enhance the likelihood of a successful bony fusion. ^( 1 , 9 , 16 , 17 )^

The goal of this study was to compare the biomechanical stability of these two constructs, to investigate whether dorsal-to-plantar screw orientation or plantar-to-dorsal screw orientation creates a more stable construct. The techniques were compared using two screws for fixation. From dorsal to plantar, two 32mm partially-threaded, 7.3mm cannulated cancellous screws were used. From plantar to dorsal, two 7.3mm cannulated cancellous screws were used; the short-threaded 16mm screw was used to gain compression in combination with a long-threaded screw to maximize the fixation with this approach.

## OBJECTIVE

To compare screw fixation strength for subtalar arthrodesis.

## METHODS

Eight matched-pair fresh-frozen human cadaver feet were tested for this study at the American Sports Medicine Institute (ASMI), from May to July 2018. This project has exemption from ethics committee (www.birmingham.ac.uk/Documents/university/legal/research.pdf). The age of the donors was 56 (±6.5) years old. The specimen was stored frozen at -20 ^o^ C until the day before testing. Each specimen was thawed overnight at room temperature. All subtalar joint preparations and fixations were performed by a single surgeon.

One foot from each pair was randomly assigned to one of two groups. One group underwent screw placement in the dorsal-to-plantar orientation (DP Group) from the neck of the talus to posterior calcaneus, and the other group from plantar-to-dorsal orientation (PD Group) from posterior calcaneus to the body and neck of talus.

The subtalar joint was exposed through a 2cm curvilinear incision starting from the tip of the lateral malleolus, extending distally and anteriorly up to sinus tarsi and then extending distally around 1cm. The extensor digitorum brevis muscle was elevated along with sinus tarsi fat pad in single thick flap. Cartilage from the subtalar joint was denuded both from the talus and the calcaneus.

For dorsal-to-plantar screw fixation, an approximately 3cm incision was made between the tibialis anterior tendon and extensor hallucis longus tendon, starting at the level of the anterior ankle joint and extending distally. The neck, medial, and lateral boundaries of the talus were exposed. The heel was held in the center of the palm, maintaining 5° of valgus at the subtalar joint. One 4.5mm threaded guide pin for a 7.3mm screw was placed from the center of the neck of the talus directed towards the center of the palm at the posterior inferior aspect of the calcaneal tubercle, to fix the subtalar joint and compress the posterior facet. Image intensification was utilized with a sagittal and a tangential view of the calcaneus to confirm accurate placement of the pin. Another pin was placed from the medial aspect of the talus neck and directed laterally and plantar towards the calcaneus to fix the anterior facet of subtalar joint. Drilling of the talus and superior surface of the calcaneus was carried out after determining the appropriate depth with the gauge. A 7.3mm, 32mm thread length cannulated cancellous lag screw was inserted over the pin to tightness. A second guide pin was placed distally and more medially on the talar neck, directed more laterally and plantar into the anterior process of the calcaneus. The second pin was measured and overdrilled and the screw was inserted.

For plantar-to-dorsal screw fixation, an approximately 2cm incision was made posteroinferiorly over the calcaneus distal to the Achilles tendon insertion. Two 4.5mm threaded guide pins were placed in parallel from the posterior aspect of calcaneus into the body and neck of the talus. Two 7.3mm cannulated cancellous screws were placed over the guide pins after drilling across the plantar cortex of the talus. Placement of the screws was confirmed with image intensification, using sagittal and anterior views of the ankle and a tangential view of the calcaneus.

**Figure 1:**
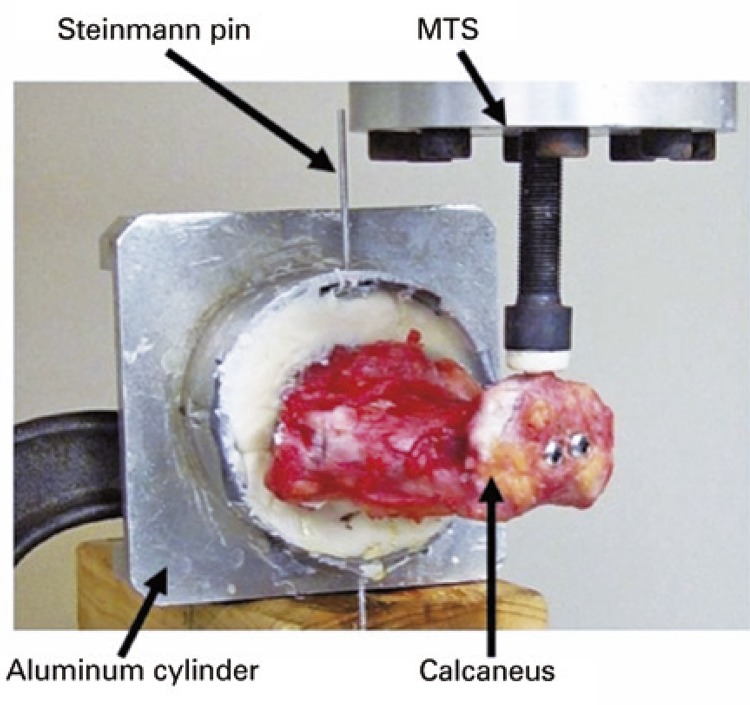
Experimental setup. The talus is potted within the aluminum cylinder. A Steinmann pin is drilled to hold the talus in place. The medial aspects of the talus and calcaneus are facing up MTS: Bionix ^®^ 858 MTS.

**Figure 2:**
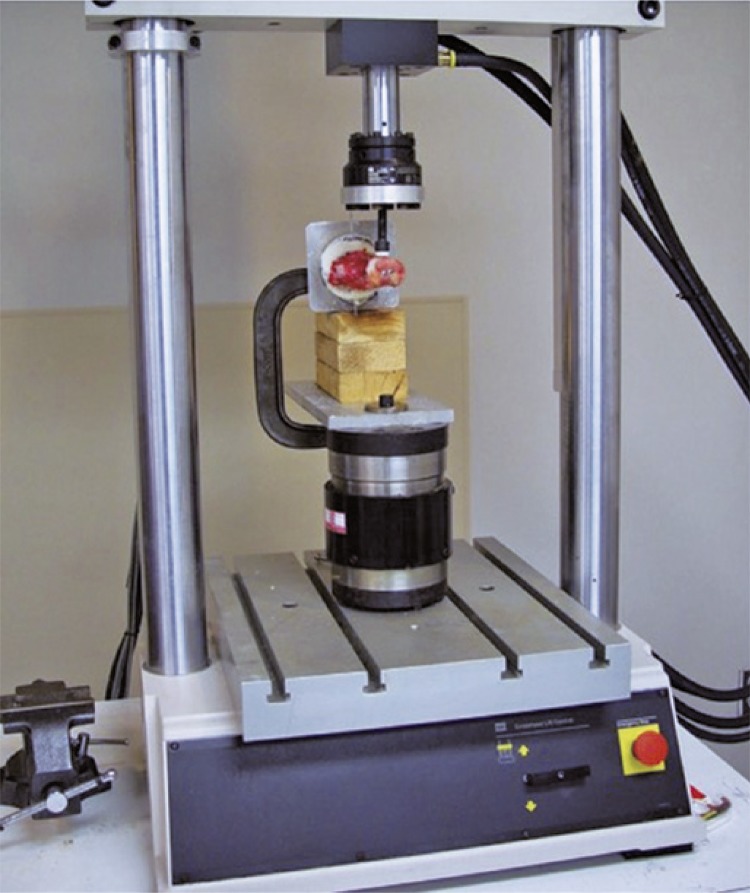
Experimental setup. The construct was mounted on a material testing system

After all the soft tissues were removed from the bones, the talus was potted into an aluminum cylinder using polymethyl-methacrylate (PMMA) ( Figure 1 ). A threaded Steinmann pin was drilled through the holes on the cylinder wall, PMMA, and talus to help hold the specimen in position. The construct was mounted on a material testing system (Bionix ^®^ 858, MTS, Eden Prairie, MN) with the talus and calcaneus in the horizontal direction and the medial aspects of each facing up ( Figure 2 ). The position of the construct was adjusted so that the vertical load could be applied to the distal point of the medial aspect of the calcaneus, with the tip of the actuator simulating a medially-applied force at the subtalar joint. Vertical load was applied at 5mm/s. ^( 18 )^ The test was terminated when an approximately 5mm opening (measured by a tape) at the subtalar joint was observed. The force to failure was determined to be the force at which the fixation failed, which was indicated by a sudden drop in the load-displacement. ^( 6 )^ The distance between the point of force application to the center of the subtalar joint line was measured using a digital caliper (500-196-20 0-6 Digimatic Caliper, Mitutoyo America, Aurora, IL) and served as the moment arm. The product of this moment arm and the force to failure was considered as the screw fixation failure torque.

The fixation strengths were compared between the two groups using a paired *t* test. Statistical significance was kept at p<0.05. Statistical analysis was conducted using (SPSS), version 11.5.0 software (SPSS Inc., Chicago, IL).

## RESULTS

**Figure 3:**
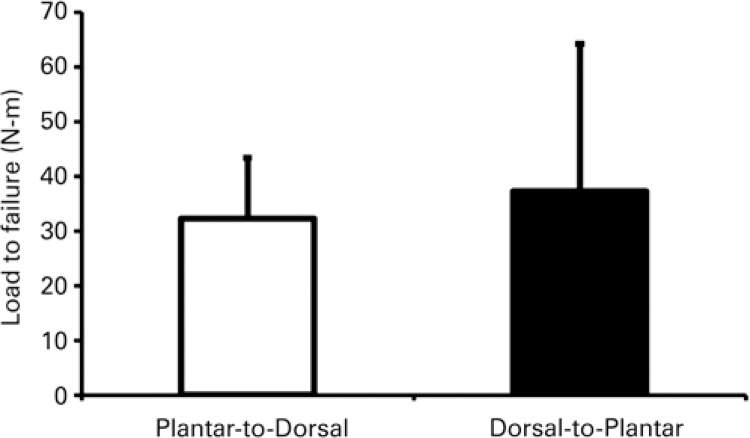
Comparison of load to failure between plantar-to-dorsal and dorsal-to-plantar fixations

**Figure 4:**
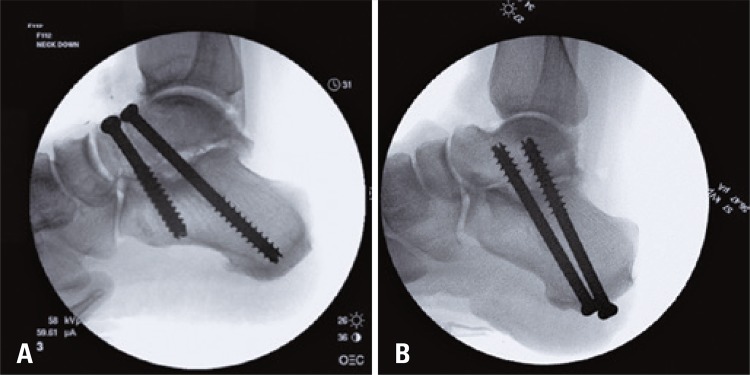
Screw placements. (A) Dorsal-to-plantar screw orientation. Two 7.3mm cannulated screws were placed from the talus, directed posteriorly through the calcaneus toward the plantar surface. (B) Plantar-to-dorsal screw orientation. Two 7.3mm cannulated screws were directed from the calcaneus, anteriorly and superiorly through the talus

The force to failure was 585.9±201.1N for the plantar-to-dorsal fixation and 667.2±449.4N for the dorsal-to-plantar fixation. The moment arm was 55.1±4.7mm for the dorsal-to-plantar fixation and 54.8±3.9mm for the plantar-to-dorsal fixation. The failure torque was 32.2±11.2Nm and 37.3±26.9Nm for the plantar-to-dorsal and dorsal-to-plantar fixations, respectively ( Figure 3 and 4 ). Even though the average failure torque of the dorsal-to-plantar technique was slightly greater than that of the plantar-to-dorsal technique, the difference was not statistically significant (p=0.55).

## DISCUSSION

When performing subtalar arthrodesis, internal fixation with screws has been shown to achieve exceptional compression and rigid immobilization. Surgeons have the choice of types of screw, number of screws, and direction of screw placement. ^( 19 )^ There is no consensus on which manner of fixation is the best. ^( 20 , 21 )^

Rate of nonunion with subtalar arthrodesis has been reported to vary between 2% and 30%. ^( 22 )^ Meticulous preparation of the joint, compression, and immobilization are especially important for preventing non-union and malunion. ^( 19 )^ The use of double parallel screws and double diverging screws have both been identified to provide support under two to three-fold greater compressive force than a single-screw construct. Chuckpaiwong et al. provided biomechanical evidence for selecting specific screw constructs, trajectories, and patterns. A double screw fixation displayed greater compression, torsional stiffness, and resistance to joint rotation. ^( 23 )^

There is also a positive correlation between bone density and compression capacity of screws. ^( 22 )^ The bone in the neck is harder than the soft cancellous bone of the heel, which gives better purchase to the head. We therefore used the long-threaded 32mm screws for better calcaneal stability. ^( 11 )^

Previous literature has not compared the biomechanical effectiveness and fusion rates between placing screw from the heel up and from the talar neck down for internal fixation. Fixation in the plantar-to-dorsal direction, from calcaneus to talus, has become routine in many practices. ^( 11 )^ This approach is commonly used when performing a calcaneal osteotomy with subtalar fusion, as in rheumatoid arthritis, severe deformity in posterior tibial dysfunction, and in some cases of arthritis secondary to calcaneal fractures. ^( 18 )^ The advantages described are that this technique has an easier initial approach, access to denser talar bone once the screw is placed, and less risk of neurovascular injury than the dorsal-to-plantar approach. ^( 16 )^ Any disturbance of bone supply can easily deprive the bone of the oxygen source, potentially leading to talar avascular necrosis. The plantar-to-dorsal technique avoids this complication. ^( 24 )^

The alleged disadvantages to the heel approach are that the calcaneal cortex is often relatively soft, necessitating use of a washer to stabilize the screw head and prevent penetration, and that the short-threaded screw is needed for at least the first screw to allow compression and avoid the threading from crossing the joint. ^( 11 )^ Additionally it is often cumbersome to the surgeon to control foot position in addition to having to hold the foot up in order to place the screws. Consistent with Kunzler et al., a frequent complaint with this technique is symptomatic hardware from prominence of headed screws, occurring from 11% to 53% of cases. The patients walk on the screws which elicits pain and the screws often need to be taken out, requiring a return to the operating room. A simple solution to this was the suggested use of headless screws that allow burial below the cortical surface. ^( 25 )^

The dorsal-to-plantar technique has its own benefits and disadvantages as well. When osteotomy of the calcaneus or other indications listed above are not present, this approach allows simple supine positioning of the patient with a bump under the hip to bring the foot to a neutral rotation position, and only two fluoroscopy views are required to check position of the pin. The surgeon can hold the foot in one hand and place the pins and screws with the drill in the other hand in an easily reproducible manner. This also allows the surgeon to manipulate the foot easily, observe the range of motion and relation between the talus and calcaneus, and assess the impact on procedure outcome. ^( 14 )^ By holding the heel in the midpoint of the palm and aiming to this point with the pin through the neck of the talus, accurate placement of this first pin can be frequently achieved on the first try. After this screw is inserted, the first guide pin can be left in place and the second pin can easily be oriented based on the first. ^( 26 )^ In contrast, placing the guide pins from plantar to dorsal requires placing the patient in the lateral position, holding that position with a device such as a vacuum “bean-bag”, making certain that the fluoroscopy beam rotation is perfectly sagittal, and acquiring three views to ensure accurate pin placement. ^( 9 )^

One limitation of the dorsal-to-plantar technique is the possibility of anterior ankle impingement on the lower anterior tibia by the hardware, if the screws are not placed deep enough in the talar neck. Limited thread tightness has also been reported in the less dense calcaneal bone. ^( 11 )^ This can be overcome by confirming the depth fluoroscopically and ensuring appropriate range of motion of the ankle after arthrodesis. The surgeon can also solve this by countersinking the screws or placing the screws further down the talar neck if the anatomy allows.

Avascular necrosis is an additional risk to take into consideration with the dorsal-to-plantar technique. It has been reported in the literature that avascular necrosis occurs at the subchondral bone, at the subtalar joint, in a sizeable portion of patients who develop this complication. Of note, the results of this study showed a large standard deviation of 449.4N for force failure in the dorsal-to-plantar fixation construct, as compared to 201.1N in the plantar-to-dorsal fixation construct. This variation may suggest that stability using the dorsal-to-plantar approach is less predictable; however, no definite conclusion can be drawn and this would warrant further investigation.

In terms of limitations for this study, sample size was small as only eight matched pairs of cadaveric limbs were included. Furthermore, only the strength of the construct was tested in the Bionix 858 MTS, ignoring the other biological factors involved in the successful arthrodesis of the subtalar joint. Additionally, the bone marrow density of the cadaveric bone was unknown, which may impact the overall strength of the constructs. Future studies that account for the factors mentioned above are warranted to compare fusion rates with the dorsal-plantar and plantar-dorsal techniques.

No significant difference was found between the dorsal-to-plantar or the plantar-to-dorsal technique with respect to the failure torque of the screw construct.

## CONCLUSION

Although no definitive conclusions can be drawn with respect to this study’s clinical implications, our results suggest that the surgeon may be able to determine the manner of fixation by taking into account additional procedures that need to be accomplished, rather than by which screw orientation will provide the most stable arthrodesis. When subtalar fusion needs to be combined with a calcaneal osteotomy, the screws can be placed from the calcaneus to the talus. If there is no need for an osteotomy, the method is at the surgeon’s discretion.
