# Supplementary material for: Biomechanical comparison of plantar-to-dorsal and dorsal-to-plantar screw fixation strength for subtalar arthrodesis
Source: Einstein (Sao Paulo). 2020 Feb 27;18:e0AO5052. doi: 10.31744/einstein_journal/2020AO5052 (PMC7046341; doi:10.31744/einstein_journal/2020AO5052)
Supplement: Translation

# Translation: Comparação biomecânica da força de fixação de parafuso com orientação de plantar para dorsal e vice-versa para artrodese subtalar

**Authors:** Nileshkumar Chaudhari, Alexandre Leme Godoy-Santos, Cesar de Cesar Netto, Ramon Rodriguez, Shouchen Dun, Jun Kit He, Haley McKissack, Glenn S. Fleisig, Eduardo Araujo Pires, Ashish Shah

**Affiliations:** 1 United States University of Alabama at Birmingham, Birmingham, AL, United States .; 2 Brasil Hospital Israelita Albert Einstein, São Paulo, SP, Brasil .; 3 Brasil Hospital das Clínicas, Faculdade de Medicina, Universidade de São Paulo, São Paulo, SP, Brasil .; 4 United States University of Iowa Health Care, Carver College of Medicine, Iowa City, Iowa, United States .; 5 United States Tulane University Orthopaedics, New Orleans, LA, United States .; 6 United States American Sports Medicine Institute, Birmingham, AL, United States .

**Keywords:** Articulação subtalar, Artrodese, Tornozelo, Instabilidade articular, Artrite, Artropatias

## Abstract

**Objetivo:**

Comparar a força de fixação dos parafusos para artrodese subtalar.

**Métodos:**

Oito pares de pés de cadáveres frescos foram submetidos à artrodese da articulação subtalar com dois parafusos canulados de 7,3mm. A randomização foi usada para atribuir a orientação do parafuso, de modo que um pé em cada par foi designado com orientação de dorsal para plantar (Grupo DP), e o outro pé com orientação de plantar para dorsal (Grupo PD). Técnica cirúrgica padrão com radioscopia foi usada para os procedimentos. Após a fixação, cada amostra foi testada até a falha com um dispositivo Bionix^®^858 MTS, aplicando força axial descendente a uma distância para criar torque. O torque de falha foi comparado entre os Grupos DP e PD, usando o teste *t* de Student, com p=0,05 usado para determinar significância estatística.

**Resultados:**

A análise estatística demonstrou que a média do torque até a falha favoreceu ligeiramente o Grupo DP (37,3Nm) em relação ao PD (32,2Nm). No entanto, a diferença entre os dois grupos não foi estatisticamente significativa (p=0,55).

**Conclusão:**

Na artrodese subtalar, não há diferença significativa na força de compressão entre as orientações dos parafusos dorsal-plantar e plantar-dorsal. A abordagem escolhida pelo cirurgião deve ser baseada em outros fatores, sem preocupação com a força biomecânica da orientação dos parafusos.

## INTRODUÇÃO

A artrodese da articulação subtalar é um tratamento eficaz para pacientes com instabilidade ou artrite subtalar isolada.^( 1 , 2 )^O desenvolvimento de artrite da articulação subtalar pode ser idiopático ou secundário a traumas,^( 3 )^artropatia inflamatória,^( 4 )^coalizão tarsal^( 5 )^e artrodese do tornozelo. A articulação subtalar pode se tornar instável por insuficiência ligamentar ou tendínea,^( 6 , 7 )^ou por disfunção neuromuscular.^( 8 )^A artrodese subtalar é feita como um componente da artrodese tripla para o tratamento de pé plano valgo adquirido do adulto em estágio III, para corrigir o desalinhamento do retropé. Porém, a artrodese subtalar isolada também é recomendada para alterações talocalcaneanas, por seu risco diminuído de artrite adjacente e de falha na consolidação ou consolidação viciosa da articulação tarsal transversa. Em todos os casos, o procedimento é realizado para aliviar a dor e melhorar a função.^( 1 , 2 , 9 )^A causa da dor, nessas alterações, são as mudanças na geometria articular e nos movimentos articulares. Existe um aumento do estresse nas articulações, e o pé é forçado a fazer movimentos anormais, o que resulta em aumento do estresse de contato e força que levam a mais degeneração.^( 10 )^O objetivo é consolidar a articulação subtalar em posição sólida e fisiológica, para evitar dores de movimento.^( 11 )^

As taxas de fusão na artrodese subtalar variam entre 84% e 100% em diversos estudos.^( 1 , 2 , 12 )^As técnicas operatórias incluem muitos adjuntos para se obter a fusão, incluindo o enxerto ósseo autógeno, aloenxerto e, mais recentemente, diversos tratamentos biológicos ( *orthobiologics* ).^( 13 )^A artrodese subtalar com enxertos ósseos autólogos presa com parafuso para osso esponjoso apresenta grande estabilidade e boa taxa de consolidação. Antes de 1970, a fixação interna não era utilizada para esse procedimento, especialmente em crianças. Inicialmente, eram usados pinos de Steinmann para manter a posição obtida da articulação; depois, passou-se a utilizar grampos cirúrgicos e dispositivos de fusão.^( 14 , 15 )^Com o advento das técnicas AO de fixação (https://www.aofoundation.org/), foram introduzidos parafusos de compressão para manter a posição e aumentar a probabilidade de sucesso da fusão óssea.^( 1 , 9 , 16 , 17 )^

Este estudo tem como objetivo comparar a estabilidade biomecânica desses dois construtos, para investigar qual orientação do parafuso cria um construto mais estável: se dorsal para plantar (DP) ou plantar para dorsal (PD). As técnicas foram comparadas utilizando-se dois parafusos para fixação. Para a orientação DP, utilizaram-se dois parafusos para ossos esponjosos canulados de 7,3mm de comprimento, com rosca de 32mm. Para a orientação PD, utilizaram-se dois parafusos para ossos esponjosos canulados de 7,3mm de comprimento, com rosca de 16mm, para ganhar compressão em combinação com parafuso de rosca longa para maximizar a fixação.

## OBJETIVO

Comparar a força de fixação do parafuso para artrodese subtalar.

## MÉTODOS

Oito pares combinados de pés de cadáveres humanos frescos foram testados para este estudo no *American Sports Medicine Institute* (ASMI), de maio a julho de 2018. Este projeto teve isenção do Comitê de Ética (www.birmingham.ac.uk/Documents/university/legal/research.pdf). A idade dos doadores era 56 (±6,5) anos. Os espécimes foram armazenados congelados a -20°C até o dia antes do teste. Cada espécime foi descongelado durante a noite em temperatura ambiente. Todas as preparações articulares subtalares e fixações foram feitas por um único cirurgião.

Um pé de cada par foi aleatoriamente designado para um dos dois grupos. Um grupo teve a colocação do parafuso feita na orientação DP (Grupo DP), do colo do tálus ao calcâneo posterior. O outro grupo teve a colocação feita na orientação PD (Grupo PD), do calcâneo posterior para o corpo e colo do tálus.

A articulação subtalar foi exposta por meio de incisão curvilínea de 2cm, da ponta do maléolo lateral, estendendo-se em sentido distal e anterior por 1cm até o seio tarso. O músculo extensor curto dos dedos foi elevado junto da bolsa de gordura do seio tarso em retalho único. A cartilagem da articulação subtalar foi desnudada do tálus e do calcâneo.

Para a fixação DP, foi feita incisão de aproximadamente 3cm entre o tendão tibial anterior e o tendão flexor longo do hálux, começando na altura da articulação do tornozelo anterior e estendendo-se em sentido distal. Foram expostos os bordos medial e lateral e o colo do tálus. O calcanhar foi segurado no centro da palma, mantendo-se 5° de valgo na articulação subtalar. Colocou-se um pino guia com rosca de 4,5mm para parafuso de 7,3mm do centro do colo do tálus em direção ao centro da palma, no aspecto inferior posterior do tubérculo do calcâneo, para ajustar a articulação subtalar e comprimir a faceta posterior. Foi utilizada intensificação de imagem, com visão sagital e tangencial do calcâneo, para confirmar a colocação correta do pino. Outro pino foi inserido do aspecto medial do colo do tálus, em sentido lateral e plantar, em direção ao calcâneo, para ajustar a faceta anterior da articulação subtalar. Foi feita a perfuração do tálus e da superfície superior do calcâneo, após determinação da profundidade adequada com o medidor. Foi inserido parafuso de osso esponjoso canulado de 7,3mm de comprimento e de rosca de 32mm no pino-guia, até que ficasse justo. Um segundo pino guia foi colocado em sentido distal e medial no colo do tálus, em direção mais lateral e plantar no processo anterior do calcâneo. O segundo pino foi medido e alargado, e o parafuso foi inserido.

Para a fixação PD, foi feita incisão de cerca 2cm posteroinferior sobre o calcâneo distal até a inserção no tendão de Aquiles. Dois pinos-guia com rosca de 4,5mm foram colocados paralelos à parte posterior do calcâneo e inseridos no corpo e colo do tálus. Dois parafusos de osso esponjoso canulados de 7,3mm foram colocados sobre os pinos-guia após perfuração por meio do córtex plantar do tálus. A colocação dos parafusos foi confirmada com intensificação de imagem, utilizando-se projeção sagital e anterior do tornozelo, e tangencial do calcâneo.

Após a remoção de todos os tecidos moles dos ossos, o tálus foi fixado em um cilindro de alumínio com polimetilmetacrilato (PMMA) ( Figura 1 ). Um pino de Steinmann rosqueado foi inserido através dos orifícios na parede do cilindro, PMMA, passando também através do tálus, para manter o espécime na mesma posição. O construto foi fixado em um sistema de testagem de materiais (Bionix^®^858, MTS, Eden Prairie, MN), com o tálus e o calcâneo em direção horizontal e suas porções mediais viradas para cima ( Figura 2 ). A posição do construto foi ajustada de forma que a carga vertical pudesse ser aplicada ao ponto distal da porção medial do calcâneo, com a ponta do atuador simulando uma aplicação de força medial na articulação subtalar. A carga vertical foi aplicada a 5mm/s.^( 18 )^O teste terminou quando foi observada uma abertura de aproximadamente 5mm (medida com fita métrica) na articulação subtalar. A força até a falha foi determinada pela força exercida, quando houve falha da fixação, indicada por queda repentina no deslocamento da carga.^( 6 )^A distância entre o ponto de aplicação de força até o centro da linha da articulação subtalar foi medida por compasso de calibre digital (500-196-20 0-6 Digimatic Caliper, Mitutoyo America, Aurora, IL) e serviu como a distância perpendicular. O produto dessa distância perpendicular e a força até a falha foi considerado como o torque de falha da fixação do parafuso.

**Figura 1:** Configuração experimental. O tálus é fixado dentro no cilindro de alumínio. Um pino de Steinmann é colocado para manter o tálus no lugar. As porções mediais do tálus e do calcâneo estão viradas para cima MTS: Bionix^®^858 MTS.

**Figura 2:** Configuração experimental. O construto foi fixado no sistema de testagem de materiais

As forças de fixação foram comparadas entre os dois grupos, utilizando-se o teste *t* pareado. A significância estatística foi mantida em p<0,05. A análise estatística foi feita com o software (SPSS), versão 11.5.0 (SPSS Inc., Chicago, IL).

## RESULTADOS

A força até a falha foi de 585,9±201,1N para a fixação PD e 667,2±449,4N para a fixação DP. A distância perpendicular foi de 55,1±4,7mm para a fixação DP e 54,8±3,9mm para a fixação PD. O torque de falha foi de 32,2±11,2Nm e 37,3±26,9Nm para as fixações PD e DP, respectivamente ( Figura 3 e 4 ). Embora a média do torque de falha da técnica DP tenha se mostrado ligeiramente mais elevada do que a média da técnica PD, essa diferença não apresentou significância estatística (p=0,55).

**Figura 3:** Comparação das cargas até a falha entre as fixações plantar para dorsal e dorsal para plantar

**Figura 4:** Posicionamento dos parafusos. (A) Orientação dorsal para plantar. Dois parafusos canulados de 7,3mm foram colocados do tálus, posteriormente pelo calcâneo, em direção à superfície plantar. (B) Orientação plantar para dorsal. Dois parafusos canulados de 7,3mm foram colocados do calcâneo em sentido anterior e superior, através do tálus

## DISCUSSÃO

Para a artrodese subtalar, a fixação interna com parafusos demonstra excelente compressão e imobilização rígida. Os cirurgiões podem escolher entre diferentes tipos e quantidade de parafusos, e a direção em que serão colocados.^( 19 )^Não há consenso sobre qual a melhor maneira de fixação.^( 20 , 21 )^

O índice de falha de consolidação em artrodese subtalar varia entre 2% e 30%.^( 22 )^A preparação meticulosa da articulação, a compressão e a imobilização são extremamente importantes para prevenir a falha da consolidação ou consolidação viciosa malunion.^( 19 )^Há evidências de que o uso de dois parafusos, paralelos ou divergentes, aumenta de duas a três vezes a resistência à força compressiva, com relação a um construto com apenas um parafuso. Chuckpaiwong et al., demonstraram evidências biomecânicas para construtos, trajetórias e padrões específicos de parafusos. A fixação com dois parafusos apresentou maior compressão, rigidez na torção e resistência à rotação articular.^( 23 )^

Há também correlação positiva entre densidade óssea e a capacidade de compressão dos parafusos.^( 22 )^O osso no colo é mais duro do que o osso esponjoso e mole do calcanhar, dando mais firmeza para a cabeça. Utilizamos, assim, o parafuso com rosca de 32mm para dar mais estabilidade ao calcanhar.^( 11 )^

Não há, na literatura, comparação entre a efetividade biomecânica e as taxas de fusão entre a colocação do parafuso do calcanhar para cima ou do colo do tálus para baixo para fixação interna. A fixação no sentido PD, do calcâneo para o tálus, tornou-se rotina em diversos casos.^( 11 )^Essa abordagem é comumente usada em osteotomias do calcâneo com fusão subtalar, como em casos de artrite reumatoide, deformidade grave em disfunção tibial posterior e em alguns casos de artrite secundária a fraturas do calcâneo.^( 18 )^As vantagens descritas incluem o fato de que essa técnica tem abordagem inicial mais fácil, dá acesso aos ossos mais densos do tálus após a inserção do parafuso e apresenta menor risco de lesão neurovascular do que a abordagem DP.^( 16 )^Qualquer perturbação do suprimento ósseo pode fazer com que o osso perca o acesso à fonte de oxigênio, potencialmente levando à necrose avascular do tálus. A técnica PD evita tal complicação.^( 24 )^

As desvantagens da técnica PD incluem o fato de que o córtex do calcâneo é, muitas vezes, relativamente mole e requer o uso de arruelas para estabilizar a cabeça do parafuso e prevenir a penetração, e, além disso, a rosca curta é necessária pelo menos no primeiro parafuso, para permitir compressão e evitar que a rosca atravesse a articulação.^( 11 )^Ademais, é extremamente incômodo para o cirurgião controlar a posição do pé, além de ter que segurá-lo levantado, para colocar os parafusos. Conforme observado também por Kunzler et al., uma queixa frequente com essa técnica é a proeminência sintomática da cabeça dos parafusos, o que ocorre em 11% a 53% dos casos. Os pacientes andam sobre os parafusos, o que causa dor e, muitas vezes, requer a remoção dos parafusos, bem como nova cirurgia. Um solução simples é o uso de parafusos sem cabeça, o que permite que eles sejam colocados abaixo da superfície cortical.^( 25 )^

A técnica DP tem seus próprios benefícios e desvantagens. Quando a osteotomia do calcâneo ou outras indicações listadas aqui não estão presentes, essa abordagem permite que o paciente fique em posição supina com elevação na área do quadril, de forma a trazer o pé para uma posição de rotação neutra; além disso, requer apenas duas imagens de fluoroscopia, para checar a posição do pino. O cirurgião pode segurar o pé com uma mão e colocar os pinos e parafusos com a broca cirúrgica com a outra mão, de forma facilmente reprodutível. Isso também permite que o cirurgião manipule o pé com facilidade, observe a amplitude de movimento e a relação entre o tálus e o calcâneo, e faça avaliações sobre impacto no desfecho do procedimento.^( 14 )^Segurando-se o calcanhar com o ponto central da palma e utilizando esse ponto como mira para o pino em direção ao colo do tálus, a colocação precisa desse primeiro pino é geralmente obtida na primeira tentativa. Após a inserção do parafuso, o primeiro pino-guia pode ser deixado no lugar, e o segundo pino é facilmente guiado com base no primeiro.^( 26 )^Em contraste, a colocação dos pinos-guia no sentido PD requer que o paciente esteja em posição lateral, o que demanda colchão posicionador a vácuo, sendo necessário garantir que a rotação dos raios de fluoroscopia seja precisamente sagital, com três imagens para assegurar a colocação precisa do pino.^( 9 )^

Uma limitação da técnica DP é a possibilidade de impacto do tornozelo anterior na tíbia anterior inferior, devido aos parafusos, caso os parafusos não tenham penetrado o suficiente no colo do tálus. Também há relatos de firmeza limitada na rosca dos parafusos em ossos do calcâneo com menor densidade.^( 11 )^Isso pode ser resolvido, confirmando-se a profundidade, por meio de fluoroscopia e garantindo que haja amplitude adequada de movimento do tornozelo após a artrodese. O cirurgião também pode resolver essa questão, escareando os parafusos ou os colocando mais fundo no colo do tálus, se a anatomia permitir.

A necrose avascular é um risco adicional a ser levado em conta na técnica DP. Há relatos na literatura da ocorrência de necrose avascular no osso subcondral, na articulação subtalar, em grande parte dos pacientes que desenvolvem tal complicação. Vale destacar que os resultados deste estudo mostraram grande desvio padrão de 449,4N para força de falha na fixação DP, em comparação a 201,1N na fixação PD. Essa variação pode sugerir que a estabilidade seja menos previsível na abordagem DP – porém, não se pode obter conclusões definitivas e são necessárias futuras investigações.

Com relação às limitações deste estudo, a amostra era pequena – apenas oito pares de membros cadavéricos foram incluídos. Além disso, foi testada apenas a força do construto no Bionix 858 MTS, não incluindo outros fatores biológicos envolvidos no sucesso da artrodese da articulação subtalar. Ademais, a densidade da medula óssea dos cadáveres era desconhecida, o que pode impactar na força geral dos construtos. São necessários estudos futuros que incluam os fatores mencionados acima para comparar os índices de fusão com as técnicas DP e PD.

Não foi observada diferença significativa entre as técnicas DP e PD com relação ao torque de falha dos construtos dos parafusos.

## CONCLUSÃO

Embora não seja possível tirar conclusões definitivas com relação às implicações clínicas deste estudo, nossos resultados sugerem que talvez o cirurgião possa determinar a maneira de fixação adequada, levando em conta os procedimentos adicionais necessários, em vez de se basear em qual orientação do parafuso resultará na artrodese mais estável. Quando a fusão subtalar precisa ser combinada com a osteotomia do calcâneo, os parafusos podem ser colocados do calcâneo para o tálus. Se não houver necessidade de osteotomia, o cirurgião pode definir o método de sua preferência.
